# Reassessing the role of the NLRP3 inflammasome during pathogenic influenza A virus infection via temporal inhibition

**DOI:** 10.1038/srep27912

**Published:** 2016-06-10

**Authors:** Michelle D. Tate, James D. H. Ong, Jennifer K. Dowling, Julie L. McAuley, Avril B. Robertson, Eicke Latz, Grant R. Drummond, Matthew A. Cooper, Paul J. Hertzog, Ashley Mansell

**Affiliations:** 1 Centre for Innate Immunity and Infectious Diseases, Hudson Institute of Medical Research, Clayton, Victoria, Australia; 2 Department of Molecular and Translational Sciences, Monash University, Clayton, Victoria, Australia; 3 Department of Microbiology and Immunology at the Peter Doherty Institute for Infection and Immunity, University of Melbourne, Parkville, Victoria, Australia; 4 Institute for Molecular Bioscience, University of Queensland, Brisbane, Australia; 5 Institute of Innate Immunity, University Hospital, University of Bonn, Bonn, Germany; 6 Department of Infectious Diseases and Immunology, University of Massachusetts Medical School, Worcester, Massachusetts, USA; 7 German Center for Neurodegenerative Diseases, Bonn, Germany; 8 Department of Pharmacology, Monash University, Clayton, Victoria, Australia

## Abstract

The inflammasome NLRP3 is activated by pathogen associated molecular patterns (PAMPs) during infection, including RNA and proteins from influenza A virus (IAV). However, chronic activation by danger associated molecular patterns (DAMPs) can be deleterious to the host. We show that blocking NLRP3 activation can be either protective or detrimental at different stages of lethal influenza A virus (IAV). Administration of the specific NLRP3 inhibitor MCC950 to mice from one day following IAV challenge resulted in hypersusceptibility to lethality. In contrast, delaying treatment with MCC950 until the height of disease (a more likely clinical scenario) significantly protected mice from severe and highly virulent IAV-induced disease. These findings identify for the first time that NLRP3 plays a detrimental role later in infection, contributing to IAV pathogenesis through increased cytokine production and lung cellular infiltrates. These studies also provide the first evidence identifying NLRP3 inhibition as a novel therapeutic target to reduce IAV disease severity.

Seasonal influenza A virus (IAV) epidemics are a major cause of morbidity and severe illness globally. The emergence of highly pathogenic H5N1 and H7N9 avian IAV strains predominately across Asia has caused sporadic infections in humans with high mortality. The potential of these stains to adapt and mediate human-to-human transmission and to cause a pandemic poses a constant and realistic threat to global health^1^, particularly as many isolates have been identified to carry genetic modifications that confer antiviral drug resistance. A characteristic feature of these deadly infections is exaggerated cellular influx into the lungs and elevated concentrations of inflammatory mediators linked to hypercytokinemia, or ‘cytokine storm’, which is indicative of poor disease outcomes^2^. Understanding the mechanisms involved in the development of a cytokine storm and fatal disease is imperative to allow for the development of improved and better-targeted treatments to reduce mortality associated with pathogenic IAV.

The NLRP3 inflammasome is an oligomeric innate immune intracellular signalling complex that senses a range of pathogen-, host- and environmental-derived factors^3^. Following activation, NLRP3 binds to the adaptor protein, apoptosis-associated speck-like protein containing a CARD (ASC). ASC further recruits the enzyme caspase-1 to form the inflammasome complex initiating autocatalytic cleavage of caspase-1. The NLRP3 inflammasome is now recognized as a major pathway by which the innate immune system recognizes and responds during IAV infection^3^. To date, IAV single-stranded RNA (ssRNA) and proton flux via the IAV-encoded matrix-2 (M2) ion channel have been shown to activate the NLRP3 inflammasome^4^,^5^, which is important in the development of adaptive immune responses to IAV^6^.

Studies utilising mice lacking components of the NLRP3 inflammasome have demonstrated its importance in eliciting an early, protective immune response when challenged with the mouse-adapted A/Puerto Rico/8/34 strain (PR8, H1N1)^5^,^7^. In these studies, inflammasome-deficient mice were highly susceptible to both low and high inoculum doses of PR8 and the NLRP3 inflammasome was shown to be required for production of pyrogenic IL-1β and IL-18 in the airways, cellular infiltration and lung immunopathology. Previously studies had shown that mice deficient in the IL-1R display increased mortality following infection with PR8 H1N1 despite a reduction in lung immunopathology^8^. Furthermore, IL-18-deficient mice also demonstrated increased mortality to A/PR8 challenge with increased pathophysiology in the lung for the first 3 days of infection, which included pronounced virus growth with massive infiltration of inflammatory cells and elevated nitric oxide production^9^.

We recently demonstrated that the IAV virulence factor PB1-F2, derived from highly pathogenic strains, activates the NLRP3 inflammasome inducing IL-1β production and pulmonary inflammation^10^. PB1-F2 also triggers excessive cellular recruitment and hyper-inflammatory responses in the lungs of infected mice^11^,^12^ and the deletion of PB1-F2 is protective to PR8-induced lethality. These findings therefore suggest that while seasonal IAV infections, factors such as M2 and viral RNA may induce protective inflammasome activation, during pathogenic IAV infections, PB1-F2-induced activation of the inflammasome induces a damaging environment to the host which can lead to the induction of disease and mortality.

In this study, we have used a small molecule inhibitor of the NLRP3 inflammasome to examine the temporal contribution of the NLRP3 inflammasome to the pathogenesis of IAV. MCC950 was first identified amongst a screen of diarylsulfonylurea-containing compounds described as novel IL-1β maturation inhibitors^13^. Recently, MCC950 was characterized as a highly specific inhibitor, blocking NLRP3, but not Toll-like receptor, AIM2, NLRC4 or NLRP1 inflammasome activation and inflammation^14^. Importantly from a clinical and specificity perspective, MCC950 blocked IL-1β secretion from PBMCs from individuals with Muckle-Wells Syndrome, a cryopyrin-associated periodic syndrome disease caused by a gain-of-function mutation in NLRP3^15^. Recently, we further utilised MCC950 to successfully reduce inflammation and blood pressure in a mouse model of hypertension^16^. These studies highlight the specificity of MCC950 and its superiority to antibody-based therapies by allowing temporal application (and if necessary removal) while avoiding the universal suppression of IL-1β secretion by other inflammasome complexes.

We have shown that early inhibition of NLRP3 inflammasome exacerbates disease consistent with the protective role previously described using inflammasome-deficient mice. In contrast, late inhibition of the NLRP3 inflammasome by MCC950 alleviated disease and reduced lung inflammation, demonstrating that the NLRP3 inflammasome was contributing to development of a ‘cytokine storm’ and lethality. Temporal inhibition of NLRP3 function therefore discloses for the first time that NLRP3 plays a dual role during pathogenic IAV: an early inflammatory response inducing a protective environment, and a subsequent excessive damaging inflammatory response that contributes to pathogenesis and mortality. This study identifies for the first time that activation of the inflammasome in the later stages of IAV disease detrimentally contributes to pathogenesis and suggests that targeting the inflammasome may be a therapeutic option to treat pathogenic IAV infections.

## Results

**Figure 1:**
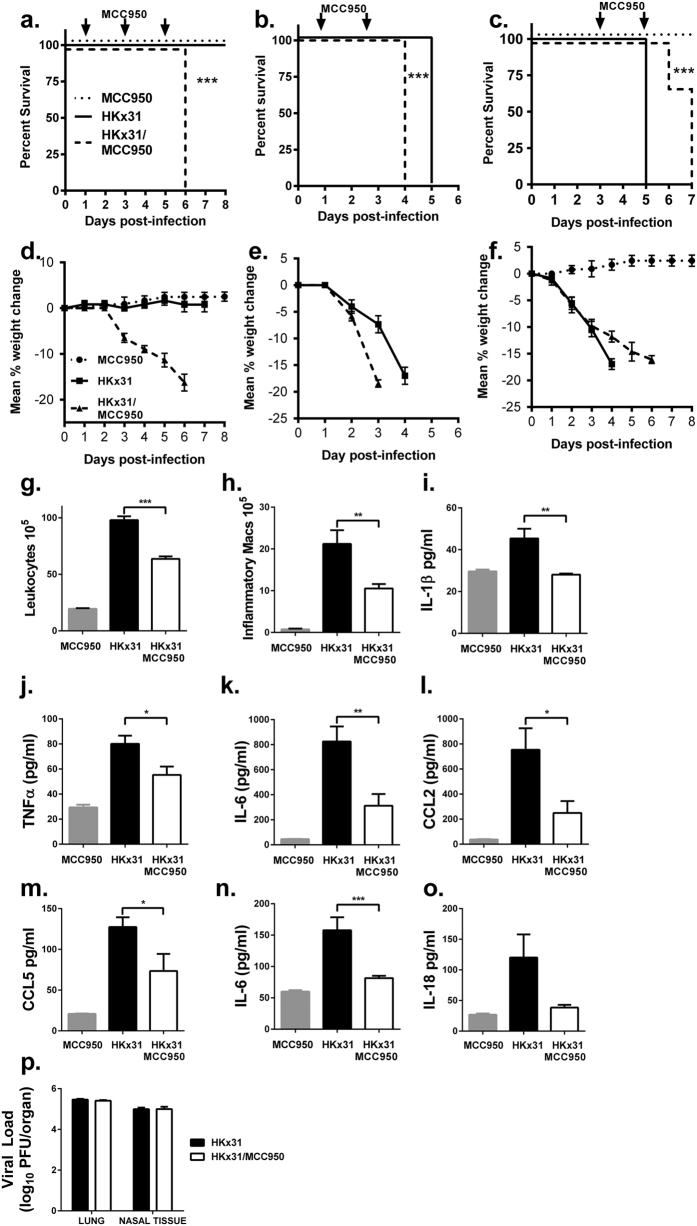
Administration of MCC950 modulates survival and reduces airway inflammation during IAV- infection. Groups of wild-type C57BL/6 mice were infected intranasally with **(a/d)** low dose (10^2 ^PFU) (n = 5/group) or **(b/c/e/f)** high dose (n = 10 per group) HKx31 (10^5 ^PFU). Mice were treated intranasally with MCC950 (5 mg/kg) at indicated time points following infection (arrows). Uninfected mice treated with MCC950 are included for comparison. **(a–c)** Survival curves are shown. ***P < 0.001, Mantel–Cox log-rank test. **(d–f)** Mice were weighed daily and resulted expressed as mean percent weight change. **(g–p)** Mice (n = 5 mice/group) were treated with MCC950 on day 3 following high dose HKx31 infection and 24 hours later **(g,h)** total numbers of leukocytes in BAL were determined by viable cell counts and Ly6G^+^ neutrophils, total CD11c^+^ MHC Class II^lo^ macrophages and Ly6C^+^ inflammatory macrophages in BAL were determined by flow cytometry. Pro-inflammatory cytokine levels were determined by ELISA or CBA in **(i–m)** BAL fluid and **(n,o)** sera. Data presented is mean ± SEM from 5 mice per group. *p < 0.05, **p < 0.01, ***p < 0.001, One-way ANOVA. **(p)** Viral loads in the lung and nasal tissues by standard plaque assay.

Using MCC950, we examined the temporal role of NLRP3-induced inflammation during mild (10^2 ^PFU; Fig. 1a,d) and severe (10^5 ^PFU; Fig. 1b,c,e and f) IAV infection. HKx31 (H3N2) is an IAV reassortant of PR8 that expresses the hemagglutinin (HA) and neuraminidase (NA) of a H3N2 human isolate from the deadly Hong Kong pandemic of 1968. Unlike the mouse-adapted PR8 strain, HKx31 is similar to many pathogenic IAV strains such as H5N1 and H7N9 in its ability to infect macrophages^17^,^18^,^19^. Mice were intranasally treated with MCC950 or PBS alone on days 1, 3 and 5 post-IAV infection. Consistent with an early protective role of NLRP3 reported previously^5^,^7^ commencement of MCC950-treatment within 24 h post-infection with either low or high doses of HKx31, resulted in mice displaying accelerated weight loss (Fig. 1d,e) and mortality (Fig. 1a,b) respectively. By comparison, uninfected mice treated with MCC950 did not display any adverse effects over an 8-day period (Fig. 1d).

We next examined the effect of delaying NLRP3 inflammasome inhibition to the later stages of HKx31 infection. Mice were infected with a high dose of HKx31 (10^5 ^PFU) to induce disease and treated with MCC950 or PBS alone on days 3 and 5, the period during which viral load in the lungs and overt signs of illness peak. As expected, HKx31-infected mice exhibited significant weight loss (Fig. 1f) and mortality within 4 days (Fig. 1c). While MCC950-treated mice also lost weight, there was a significant (48h) delay in HKx31-associated lethality (p < 0.001). Significantly, MCC950 treatment on day 3 post-HKx31 infection reduced total leukocytes and inflammatory macrophage populations in bronchoalveolar lavage (BAL; Fig. 1g,h), while neutrophils, DC infiltrates and resident macrophages were not significantly altered between the groups on day 4 post-infection (Supplementary Fig. 1a–c). Critically, on day 4 post-infection, IL-1β, TNFα, IL-6, CCL2 and CCL5 concentrations were significantly reduced in BAL fluid (Fig. 1i–m) while IL-6 and IL-18 were reduced in the sera (Fig. 1n,o). Expression of IL-12p70, IFNγ, IL-10 and CXCL1 were not altered in the lung BAL, while IL-1β, TNFα, CCL2, IFNγ, IL-12p70 and IL-10 levels in the serum were not affected (Supplemental Fig. 1e–n). Importantly, MCC950 treatment on day 3 did not alter viral loads (Fig. 1p) suggesting the suppression of inflammation was not due to a MCC950-mediated reduction in viral load.

**Figure 2:**
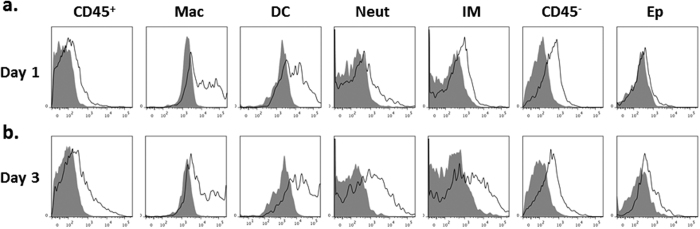
MCC950 is permissive to multiple cell types following IAV challenge. Groups of 4 wild-type C57BL/6 mice were intranasally infected with 10^4 ^PFU of HKx31 and treated with rhodamine-tagged MCC950 (MCC950-R; 10mg/kg) on days 1 or 3 post-infection. Uptake was determined 3 h following MCC950-R treatment (unshaded histograms) by flow cytometry analysis. Mice infected with HKx31 but not treated with MCC950-R were included for comparison (shaded histograms). Representative histograms for CD45^+^ leukocytes, macrophages (mac; CD11c^+^ MHC Class II^low^), dendritic cells (DC; CD11c^+^ MHC Class II^high^), neutrophils (Neut; Ly6G^+^), inflammatory macrophages (IM; Ly6C^+^ Ly6G^−^), CD45^−^ cells and epithelial cells (Ep; CD45^−^ intracellular cytokeratin C^+^).

Given MCC950 was successful in suppressing lung inflammation following viral challenge, we next wished to identify within which cells MCC950 may be mediating its inhibitory effect. We therefore treated mice with MCC950 linked to fluorescent rhodamine (MCC950-R) on days 1 or 3 post-HKx31 infection. As can be seen in Fig. 2, MCC950-R was readily detected 3 hours following intranasal treatment in a broad range of CD45^−^ (such as epithelial cells) and CD45^+^ cell types (e.g. macrophages and DCs), suggesting its absorption is non-specific.

**Figure 3:**
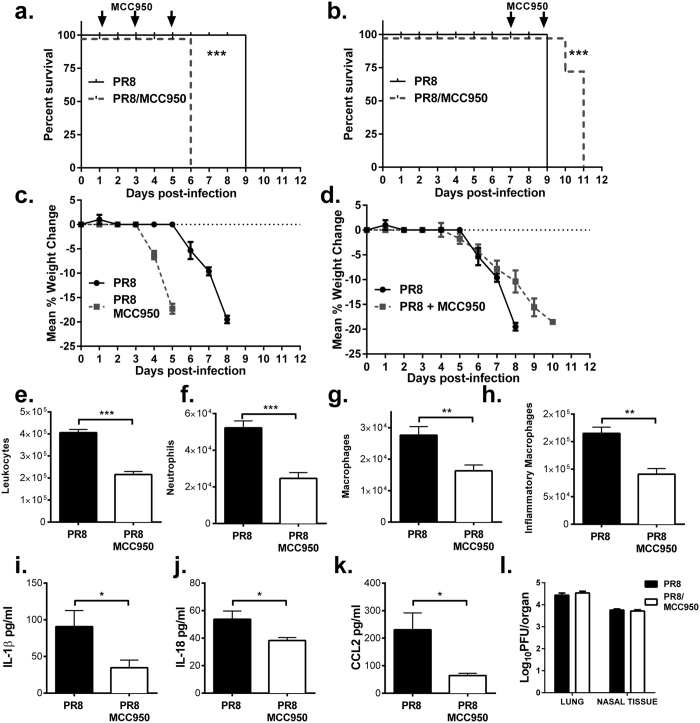
Inflammasome inhibition with MCC950 provides protection and reduces inflammation against IAV-induced pathogenicity. C57BL/6 mice (n = 5 mice/group) were intranasally infected with 50 PFU of PR8 and treated with MCC950 (5mg/kg) on **(a/c)** days 1, 3, 5, 7 or **(b/d)** day 7 and 9 (arrows). **(a/b)** Survival curves are shown. ***p < 0.001, Mantel–Cox log-rank test and represent 2 independent experiments. **(c/d)** Mice were weighed daily and resulted expressed as mean percent weight change. **(e–l)** Wild type C57Bl/6 mice (n = 5/group) were intranasally inoculated with PR8 (50 PFU) alone or in combination with MCC950 (5 mg/kg) on day 7 post-infection and euthanized 24 h later. **(e–h)** Total numbers of leukocytes in BAL were determined by viable cell counts and numbers of Ly6G^+^ neutrophils and Ly6C^+^ inflammatory macrophages in BAL were determined by flow cytometry. (**I–k**) Levels of pro-inflammatory cytokines were determined by ELISA or CBA in BAL fluid. Data presented is mean ± SEM from 5 mice per group of 2 independent experiments. *p < 0.05, **p < 0.01, ***p < 0.001, One-way ANOVA. **(l)** Viral loads in the lung and nasal tissues by standard plaque assay.

Having established that delayed administration of MCC950 prolonged survival and reduced inflammatory cell infiltrates in mice following infection with the clinically-relevant IAV HKx31, we next assessed the impact of inhibition of NLRP3 inflammasome activity against disease parameters in mice infected with the highly virulent and mouse-adapted PR8 H1N1 strain. Consistent with previous studies in inflammasome-deficient mice^5^,^7^, inhibition of NLRP3 function via treatment with MCC950 on days 1, 3 and 5 following lethal PR8 infection rendered mice hyper-susceptible, displaying significant mortality (Fig. 3a) and weight loss (Fig. 3c). Critically, however, initiation of MCC950 treatment of PR8-infected mice on day 7 provided significant protection from PR8-induced lethality (Fig. 3b), weight loss (Fig. 3d). We further assessed airway cellular infiltration and cytokine concentrations following MCC950 treatment on day 7. As per Fig. 3e–h, MCC950-treated mice displayed significantly reduced total leukocytes, neutrophils and macrophages infiltrates following PR8 infection compared to PBS-treated mice, while DC infiltrates were also reduced (Supplementary Fig. 2a). In addition, MCC950 treatment reduced B220+, CD4+ and CD8+ cells, as well as influenza-specific CD8+ T cell numbers (Supplementary Fig. 3). Importantly, BAL concentrations of inflammasome-dependent inflammatory cytokines IL-1β and IL-18 and the CCL2 were significantly reduced in MCC950-treated mice (Fig. 3i–k). Consistent with earlier results, MCC950 had no effect on viral load (Fig. 3l). In contrast to the HKx31 infection model, concentrations of IL-6, TNFα and CCL5 in BAL (Supplementary Fig. 2) were unchanged by inflammasome inhibition during PR8 infection, differences which may reflect the cellular tropism of each virus^20^ and the timing of the MCC950 treatment and analysis following infection.

## Discussion

The strength of the innate immune response to IAV infection is a key determinant in clinical outcome. Excessive inflammation can lead to death, particularly in the case of highly pathogenic IAV infections. However, the underlying mechanisms that incite excessive cellular influx into the lungs and cytokine storm are poorly understood. Previous studies using gene-deficient mice identified a protective role for the NLRP3 inflammasome during PR8 infection^5^,^7^. In contrast, we demonstrated that expression of full length PB1-F2 found in pathogenic IAV contributes to pathogenesis via activation of the NLRP3 inflammasome inducing excessive cytokine production^10^. For the first time, temporal use of the specific NLRP3 inhibitor MCC950 has identified that the NLRP3 inflammasome mediates both an early protective immune response, and later in infection, induces a highly inflammatory, damaging state that contributes to disease pathogenesis.

The emerging potential of H7N9 avian IAV to infect and cause death in humans is a very real threat, with a 38% mortality rate in hospitalized humans observed^21^. A recent study that sequenced the complete genomes of 438 H7N9, 263 related influenza related viruses and the 19 H7N9 human isolates, found promiscuous RNA swapping among H7N9 circulating in humans and avian viruses^1^. This study concluded that it is reasonable to expect the H7N9 and other viruses studied to persist and cause substantial severe human infections and that H7N9 viruses should be considered a major candidate to cause a pandemic in humans. Considering the potentially disastrous consequences of a highly pathogenic pandemic outbreak, identifying new therapeutics that target hyperinflammatory responses of the host is of urgent priority.

It is well known that the three pandemics of the 20^th^ century caused millions of deaths worldwide. While a large proportion of the fatalities have been contributed to complications arising from secondary bacterial infections^22^, the initial infection by the novel H1N1, H2N2 and H3N2 viruses caused remarkable inflammatory disease and contributed significantly to hospitalization of patients presenting with pneumonia-like illness. Recent reports analysing the clinical outcomes and hypercytokinemia of H7N9-infected patients identified that elevated cytokine levels including both IL-1β and IL-18 in BAL fluid were predictive of fatal outcomes^23^,^24^. In these studies, inflammatory cytokines such as CCL2, IL-6, IL-8, CCL4 and TNFα were high in the lung and plasma of H7N9-infected individuals, while measurable levels of IL-1β were only detected in BAL. We note that MCC950 significantly reduced the concentration of these inflammatory cytokines in our *in vivo* models. Interestingly, a study by Katz and co-workers examining the early and sustained innate response of macaques to H5N1 IAV observed a dramatic increase of acute genes such as IL-1, IL-6 and TNFα in the first 3 days following infection^25^. However, while a slight decline in induction was noted on day 4, this rebounded on day 7 to levels similar to that early in infection. This suggests there may be two ‘waves’ of inflammation further supporting the concept that excessive or sustained IL-1β and IL-18 maturation by the inflammasome may drive a ‘feed-forward’ inflammatory loop. This activation may potently activate NF-κB inducing a milieu of inflammatory cytokines and chemokines in addition to ‘priming’ the inflammasome leading to poor clinical outcomes. It is important to note that H7N9, along with PR8 and other avian IAV strains, expresses a full length PB1-F2 protein which we have found to activate the NLRP3 inflammasome in a similar manner to the PB1-F2 protein from PR8 (unpublished findings).

Recent developments and characterization of the role innate immune sensors plays in excessive inflammation has led to the emergence of therapeutics to target these sensors rather than anti-virals to treat pathogenic viral infections^26^,^27^. Indeed, two recent studies by Vogel and colleagues have ‘repurposed’ the specific TLR4 antagonist Eritoran to protect mice from pathogenic PR8 influenza infection and propose a host-targeted therapeutic approach as a novel strategy for IAV infection^28^,^29^. Critically, these studies found that the timing of Eritoran treatment was crucial for protection as early or prophylactic treatment was not protective from lethality and that a late-acting non-TLR4 mediator of lethality must be the target. These studies would therefore appear to support and enhance our findings that delayed, excessive inflammation mediated by the NLRP3 inflammasome contributes to lethality and augurs the possibility of complementary therapies to protect from the damaging inflammation characteristic of pathogenic IAV strains.

Timing of therapeutic administration is therefore critical to clinical benefit. As previously established, the NLRP3 inflammasome is crucial to establishing a protective environment^5^,^7^. Indeed, IAV components common to all strains such as IAV RNA and the M2 ion channel protein that are NLRP3 inflammasome activators^4^,^7^ which during infection may be responsible for initiating this ‘protective’ inflammasome activation, leading to protective immunity, increased disease tolerance through cellular recruitment, and induction of tissue repair^3^,^4^,^6^. However, virulence factors such as full length PB1-F2 found in avian and pandemic strains may initiate this ‘second wave’ of inflammation that drives a detrimental, pathophysiological inflammatory component. Importantly, secondary bacterial infection is also a leading cause of death during severe IAV infection and has been reported in human cases of H7N9^30^. Induction of inflammasome-driven pyroptosis may therefore lead to removal of alveolar macrophages and epithelial cell barriers which may result in increased susceptibility to secondary bacterial infections causing potentially TLR4-mediated inflammation and pneumonia^31^. Consistent with our model, we have previously demonstrated that PR8 or 1918 pandemic PB1-F2 proteins induce inflammatory disease which predisposes mice to secondary bacterial pneumonia^12^. Our study therefore, taken in conjunction TLR4 antagonism, identify that targeting these later stages of inflammation that may correspond to the clinical presentation of fulminant infection, may provide a means of reducing inflammation, pyroptosis, an environment favourable to secondary bacterial infection and reduce the cytokine and cellular influx burden. Consequently, targeting this ‘second wave’ of inflammation may only be beneficial in highly inflammatory or pathogenic IAV strains. Indeed, this and previous studies^4^,^5^,^7^ have demonstrated, early targeting of the inflammasome would presumably antagonize protective inflammation, inducing a highly susceptible immune state and ultimately not suitable for seasonal IAV infections. Therefore, determining the optimal point of intervention, or the co-administration of therapies such as TLR4 and inflammasome antagonists may provide more effective treatment for pathogenic or pandemic IAV infections.

Recent studies have further identified a critical role for the NLRP3 inflammasome in the host response to several viral and parasitic pathogens such as West Nile virus, Dengue virus, Trypanosoma cruzi and malaria (reviewed^32^,^33^). Importantly, the function and role of the NLRP3 inflammasome was characterized by use of gene-deficient mice. Our discovery that NLRP3 has both a protective and detrimental role in IAV infection and pathogenesis due to the temporal use of a specific inhibitor may suggest revisiting the role of the inflammasome in these diseases, particularly diseases such as malaria which involve periodic inflammatory episodes. While a valuable tool for characterizing gene function, our studies further highlight the limitations of relating gene function based on total gene knockout studies and emphasise the balancing role innate sensors play in inducing inflammation during disease.

Development of novel therapies that specifically target the NLRP3 inflammasome may therefore provide an effective treatment to reduce the mortality associated with pathogenic IAV, and offset the ineffectiveness of current antiviral treatments in the later stages of infection.

## Methods

### Quantification of mouse pro-inflammatory cytokines

To detect cytokines, BAL fluid and sera were collected and stored at −80 °C. IL-1β was quantified by ELISA according to manufacturer’s instructions (Becton Dickinson). Levels of IL-18 were determined by ELISA as previously described^34^. Levels of IL-6, CCL2, IFNβ, IL-10, IL-12p70, CCL5, CXCL1 and TNFα proteins were determined by cytokine bead array, mouse inflammation and flex kit (Becton Dickinson).

### Influenza virus infection of mice

6–8 week old male C57BL/6 mice were maintained in the Specific Pathogen Free Physical Containment Level 2 (PC2) Animal Research Facility at the Monash Medical Centre. All experimental procedures were approved by the Monash Medical Centre Animal Ethics Committee and experimental procedures carried out in accordance with approved guidelines. IAV strains used in this study were A/PR/8/34 (H1N1), as well as HKx31 (H3N2), which is a high-yielding reassortant of PR8 that carries the surface glycoproteins of A/Aichi/2/1968 (H3N2). Viruses were grown in 10-day embryonated chicken eggs by standard procedures and titrated on Madin-Darby Canine Kidney (MDCK) cells as described previously^35^.

For virus infection studies, groups of 10 C57BL/6 mice were anesthetized and infected with 10^2^ or 10^5 ^PFU of HKx31 (H3N2) or 50 PFU PR8 (H1N1) intranasally in 50 μl PBS. Following infection, mice were treated with MCC950 (5 mg/kg) via the intranasal route in 50 μl PBS at the time points indicated. Mice were weighed daily and assessed for visual signs of clinical disease, including inactivity, ruffled fur, laboured breathing and huddling behaviour. Animals that displayed severe clinical signs of disease were euthanized. BAL was obtained from euthanized mice via flushing the lungs three times with 1 mL of PBS. Titres of infectious virus in lung and nasal tissue homogenates were determined by standard plaque assay on MDCK cells.

### Recovery and characterization of leukocytes from mice

For flow cytometric analysis, BAL cells were treated with red blood cell lysis buffer (Sigma Aldrich, USA) and cell numbers and viability assessed via trypan blue exclusion using a hemocytometer. BAL cells were incubated with Fc block (BD Biosciences, USA), followed by staining with monoclonal antibodies to Ly6C, Ly6G, CD11c, I-A^b^ (BD Biosciences, USA). Neutrophils (Ly6G^+^), airway macrophages (CD11c^+^ I-A^d low^), dendritic cells (DC; CD11c^+^ I-A^d high^), inflammatory macrophages (IM; Ly6G^−^ Ly6C^+^) were quantified by flow cytometry, as described previously^36^. In some experiments, cells were stained with PE or APC-labelled D^b^PA_224_ (acid polymerase; SSLENFRAYV) or D^b^NP_366_ (nucleoprotein; ASNENMETM)-specific MHC-I tetramers (a gift from Department of Microbiology and Immunology, The University of Melbourne), as previously described^37^. Live cells (propidium iodide negative) were analysed using a BD FACS Canto II flow cytometer (BD Biosciences, USA) and total cell counts were calculated from viable cell counts performed via trypan blue exclusion.

### Statistical analysis

When comparing three or more sets of values, a one-way analysis of variance (ANOVA) was used with Tukey’s post-hoc analysis. A Student’s t-test was used when comparing 2 values (two-tailed, two-sample equal variance). Survival proportions were compared using the Mantel–Cox log-rank test. A *p* value < 0.05 was considered statistically significant.

## Additional Information

**How to cite this article**: Tate, M. D. *et al*. Reassessing the role of the NLRP3 inflammasome during pathogenic influenza A virus infection via temporal inhibition. *Sci. Rep.* **6**, 27912; doi: 10.1038/srep27912 (2016).

## Supplementary Material

Supplementary Information
