# Supplementary material for: Reassessing the role of the NLRP3 inflammasome during pathogenic influenza A virus infection via temporal inhibition
Source: Sci Rep. 2016 Jun 10;6:27912. doi: 10.1038/srep27912 (PMC4901306; doi:10.1038/srep27912)
Supplement: Supplementary Information [file srep27912-s1.pdf]

Supplemental Data

Reassessing the role of the NLRP3 inflammasome during pathogenic influenza A virus infection via temporal inhibition.

Michelle D Tate[1, 2], James Ong, [1, 2], Jennifer K Dowling[1, 2], Julie L McAuley[3], Avril B Robertson[4], Eicke Latz[5,6,7], Grant R Drummond[8], Matthew A Cooper[4], Paul Hertzog[1,2] and Ashley Mansell[1, 2].

[1] Centre for Innate Immunity and Infectious Diseases, Hudson Institute of Medical Research, Clayton, Victoria, Australia.

[2] Department of Molecular and Translational Sciences, Monash University, Clayton, Victoria, Australia.

[3] Department of Microbiology and Immunology at the Peter Doherty Institute for Infection and Immunity, University of Melbourne, Parkville, Victoria, Australia.

[4] Institute for Molecular Bioscience, University of Queensland, Brisbane, Australia.

[5] Institute of Innate Immunity, University Hospital, University of Bonn, Bonn, Germany.

[6] Department of Infectious Diseases and Immunology, University of Massachusetts Medical School, Worcester, Massachusetts, USA.

[7] German Center for Neurodegenerative Diseases, Bonn, Germany.

[8] Department of Pharmacology, Monash University, Clayton, Victoria, Australia

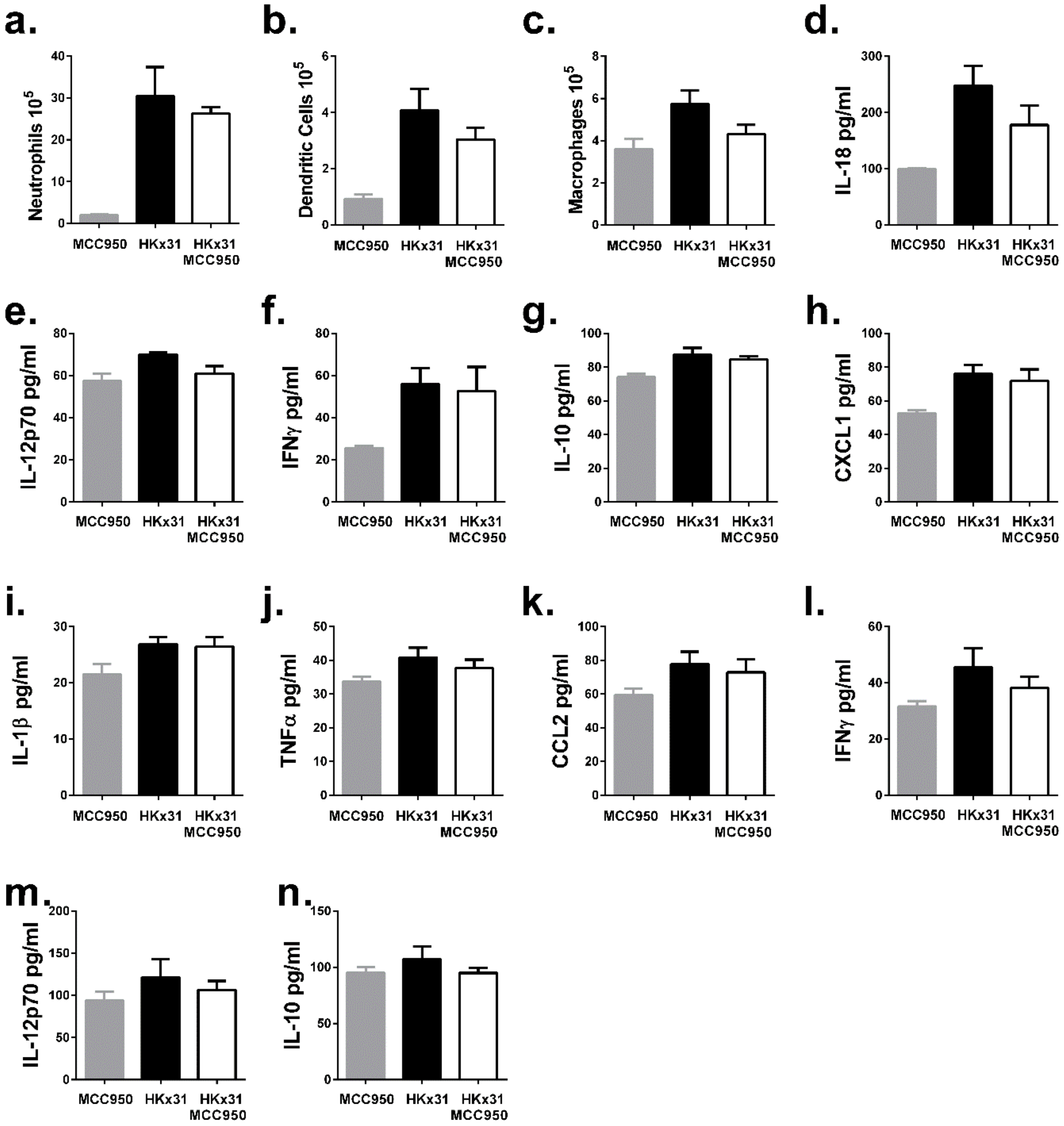

Supplemental Data 1. Inhibition of the NLRP3 inflammasome with MCC950 reduces disease severity following HKx31 infection. C57Bl/6 mice mice (n=5/group) were treated with MCC950 on day 3 following inoculation with 10^5 PFU of the HKx31 strain and 24 hours later total numbers of (**a**) Ly6G$^+$ neutrophils (**b**) CD11c$^+$ MHC Class II^{high} dendritic cells, (**c**) CD11c$^+$ MHC Class II^{low} macrophages were determined by flow cytometry. BAL (**g-h**) and sera (**i-p**) were assayed for IL-18, IL12p70, IFNγ, IL-10, CXCL1, IL-6, IL-1β, TNFα, and MCP-1 by ELISA or cytokine bead array. Results are mean ± SEM and representative of a minimum of two independent experiments

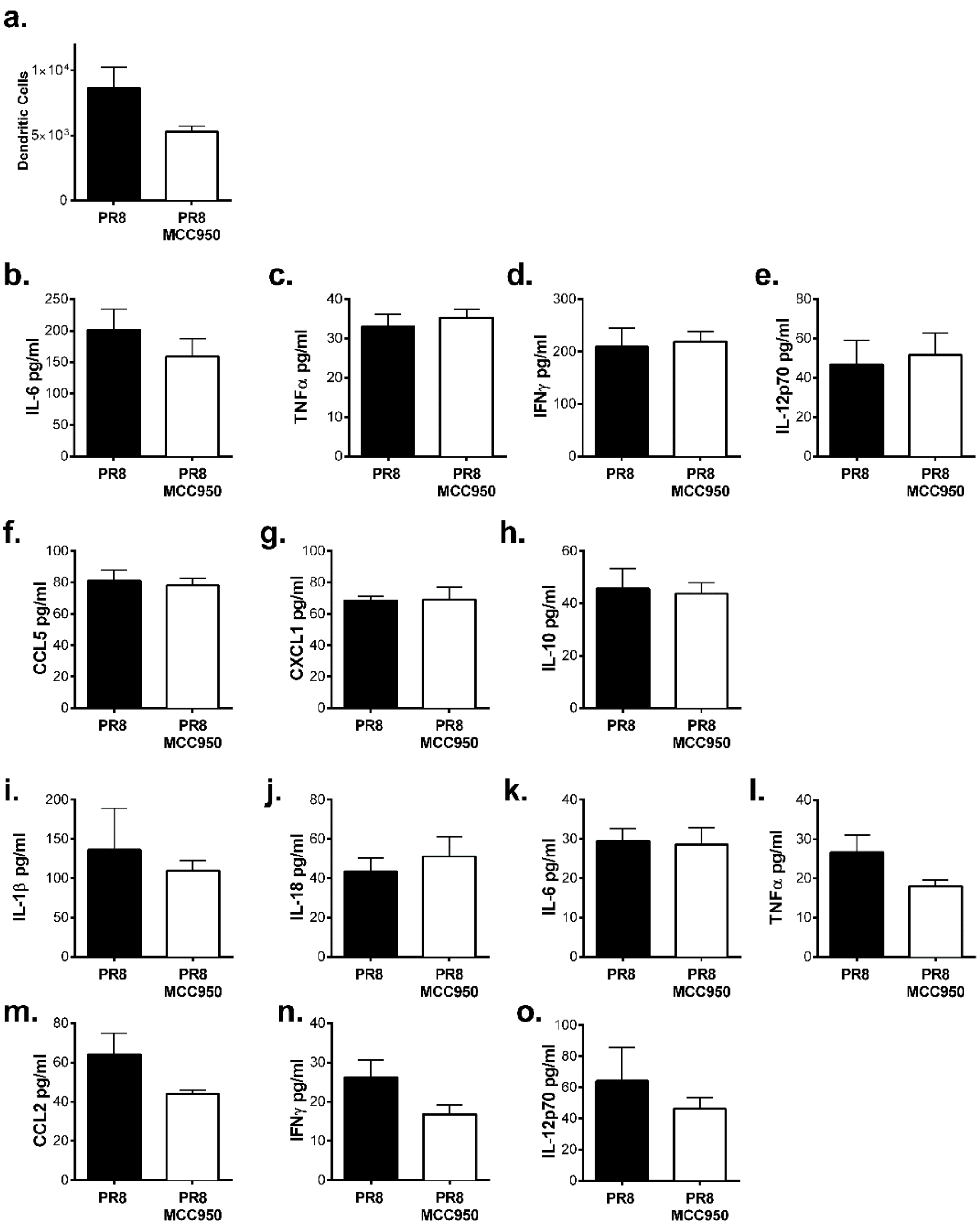

Supplemental Data 2. MCC950 treatment reduces disease severity following PR8 infection. C57Bl/6 mice were infected with 50 PFU of PR8 (n=5/group) while others were treated with MCC950 on day 7 post-infection and 24 hours later (**a**) total numbers of $CD11c^{+}$ MHC Class II^{high} dendritic cells (DC) were determined by flow cytometry. BAL fluid (**b-h**) and sera (**i-o**) were assayed for IL-6, TNFα, IFNγ, IL12p70, CCL5, CXCL1, IL-10, IL-1β, IL-18, and MCP-1 by ELISA or cytokine bead array. Results mean ± SEM and are representative of a minimum of two independent experiments.

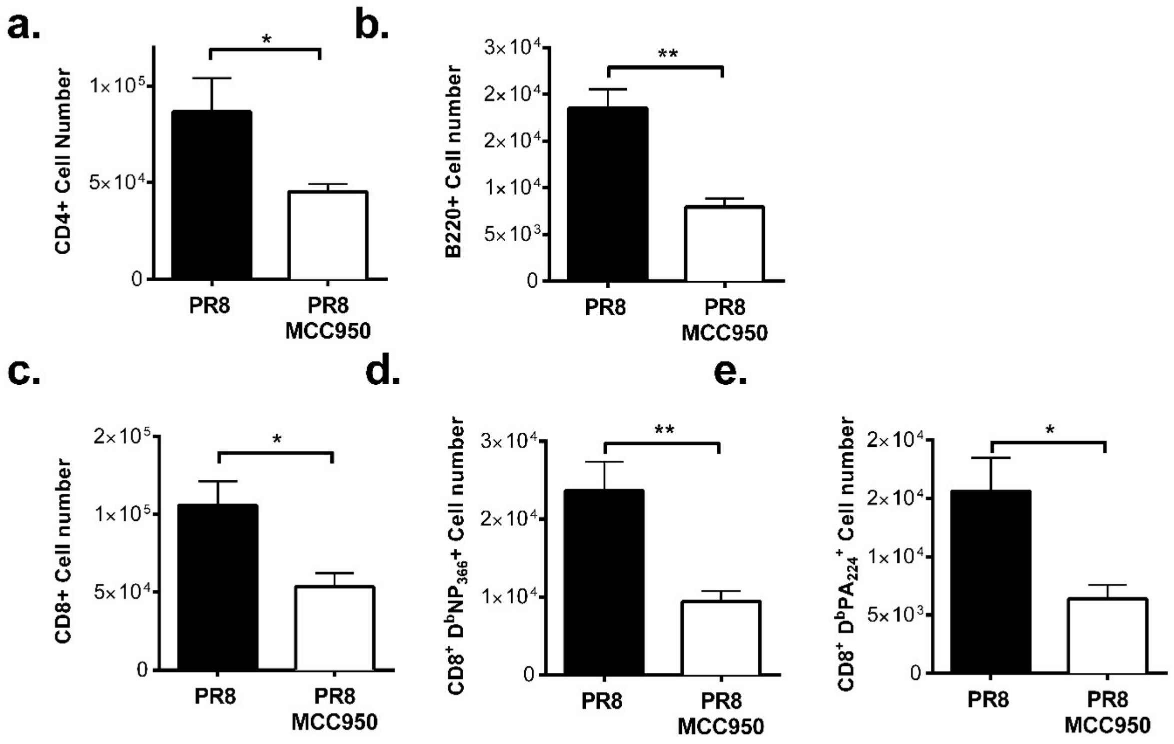

Supplementary Figure 3. MCC950-treated mice display significantly reduced adaptive responses to PR8 challenge. Wild type C57Bl/6 mice (n=5/group) were intranasally inoculated with PR8 (50 PFU) alone or in combination with MCC950 (5 mg/kg) on day 7 post-infection and euthanized 24 h later. **(a-e)** Total numbers of CD4+, B220+ and CD8+ cells as well as D^bPA_{224}-and D^bNP_{366}-specific $CD8^+$ T cells were determined by flow cytometry. Data presented is mean ± SEM from 5 mice per group of 2 independent experiments. *p<0.05, ** $p < 0.01$, One-way ANOVA.
